# Hydrogen Supplementation of Preservation Solution Improves Viability of Osteochondral Grafts

**DOI:** 10.1155/2014/109876

**Published:** 2014-11-19

**Authors:** Takuya Yamada, Kentaro Uchida, Kenji Onuma, Jun Kuzuno, Masanobu Ujihira, Gen Inoue, Bunpei Sato, Ryosuke Kurokawa, Rina Sakai, Masashi Takaso

**Affiliations:** ^1^Department of Medical Engineering and Technology, School of Allied Health Science, Kitasato University, 1-15-1 Minami-ku, Kitasato, Sagamihara, Kanagawa 252-0374, Japan; ^2^Department of Orthopedic Surgery, Kitasato University School of Medicine, 1-15-1 Minami-ku, Kitasato, Sagamihara, Kanagawa 252-0374, Japan; ^3^MiZ Co., Ltd., 1-16-5 Zenko, Fujisawa, Kanagawa 251-0871, Japan

## Abstract

Allogenic osteochondral tissue (OCT) is used for the treatment of large cartilage defects. Typically, OCTs collected during the disease-screening period are preserved at 4°C; however, the gradual reduction in cell viability during cold preservation adversely affects transplantation outcomes. Therefore, improved storage methods that maintain the cell viability of OCTs are needed to increase the availability of high-quality OCTs and improve treatment outcomes. Here, we evaluated whether long-term hydrogen delivery to preservation solution improved the viability of rat OCTs during cold preservation. Hydrogen-supplemented Dulbecco's Modified Eagles Medium (DMEM) and University of Wisconsin (UW) solution both significantly improved the cell viability of OCTs during preservation at 4°C for 21 days compared to nonsupplemented media. However, the long-term cold preservation of OCTs in DMEM containing hydrogen was associated with the most optimal maintenance of chondrocytes with respect to viability and morphology. Our findings demonstrate that OCTs preserved in DMEM supplemented with hydrogen are a promising material for the repair of large cartilage defects in the clinical setting.

## 1. Introduction

Allogenic osteochondral tissue (OCT) is one of the materials used for the treatment of large cartilage defects associated with osteonecrosis, osteochondritis dissecans, and traumatic injury due its low rates of donor-site morbidity. The transplantation of fresh OCT leads to high functional outcomes; for example, the treatment of femoral condyle defects with fresh OCTs had a greater than 75% clinical success rate [1]. However, concerns regarding the implicit risk of contamination and disease transmission associated with allogenic tissues have resulted in the implementation of a 14-day disease-screening period before OCTs are permitted for clinical use. OCTs are typically preserved at 4°C in culture medium during the disease-screening period and until they are delivered to surgeons for implantation. However, during the cold preservation of OCTs, cell viability gradually decreases, which can markedly reduce the efficacy of bone repair and transplantation treatment outcomes [2]. Therefore, preservation methods that maintain high cell viability are needed to increase the availability of high quality OCTs and improve grafting outcomes.

Numerous studies have demonstrated that the addition of fetal bovine serum (FBS) to preservation solution significantly improves chondrocyte viability in OCTs during cold storage [3–6]. Therefore, storage media for OCTs are currently supplemented with FBS. However, the United States Food and Drug Administration has raised concerns about the possibility of zoonotic and immunologic complications associated with the introduction of bovine proteins into humans. Although, to our knowledge, cross-species contamination attributable to FBS has not been reported, minimizing or eliminating the risk of potential infection associated with allogenic grafts is desirable from a regulatory and patient safety standpoint. However, because OCTs stored in serum-free culture medium at 4°C have low viability [7, 8], the development of serum-free methods for the long-term preservation of OCTs with high cell viability is needed.

One promising approach for maintaining the viability of OCTs is the supplementation of preservation solution with molecular hydrogen. The potential efficacy of hydrogen supplementation is supported by the potent antioxidant, anti-inflammatory, and antiapoptotic properties of this gas [9, 10], which was also recently reported to have strong protective effects for chondrocytes* in vitro* [11, 12]. In addition, hydrogen supplementation of organ preservation solution during cold preservation for up to 8 h reduced the severity of organ injury associated with ischemia reperfusion [13, 14]. However, the efficacy of hydrogen supplementation of storage medium for OCTs, which require markedly longer cold preservation periods than organ transplant material, has not been determined.

Hydrogen-rich solutions for the cold storage of organs are typically prepared using high-pressure hydrogen or bubbling with hydrogen [13, 15, 16]. However, these systems are susceptible to bacterial contamination and may cause mechanical injury to tissues during the bubbling process. To overcome these limitations, a nondestructive hydrogen dissolving device, consisting of an electrolyzer and cold water bath, was developed to generate and maintain hydrogen in the preservation solution at therapeutic concentrations throughout the cold storage period [14, 17]. Notably, the system does not require the storage vessel to be opened and does not alter the other ingredients in the preservation solution [17]. Therefore, this system can be safely used to supplement the preservation solution with hydrogen and potentially improve OCT viability through antioxidant and anti-inflammatory effects. Here, we evaluated whether hydrogen supplementation of preservation solution using a nondestructive hydrogen dissolver improved the cell viability of rat OCT during long-term cold preservation.

## 2. Materials and Methods

### 2.1. Preparation of OCT Samples

All procedures, including the handling of animals, were performed in accordance with the guidelines of the Animal Ethics Committee of Kitasato University. A total of 48 male 14-week-old Sprague-Dawley rats were obtained from Charles River Japan, Inc. (Yokohama, Japan). For anesthetization, the rats were first administered diethyl ether for anesthesia induction and were then fully anesthetized with a mixture of medetomidine, midazolam, and butorphanol tartrate by intramuscular injection. To excise distal femora, a skin incision was first made just above the knee. The patellar, cruciate, and collateral ligaments were cut to expose the distal femoral condyles, and the distal parts of the knee extensor muscles then were removed from the bone. Distal femora were cut at the metaphyseal region using a bone saw. The weight of OCT samples was adjusted to approximately 300 mg wet weight by trimming soft tissue and bone tissue with a scalpel or rongeur. Two OCT samples were harvested from the bilateral knees of each Sprague-Dawley rat. The 40 OCT samples obtained from 20 rats were randomly separated into 4 groups (*n* = 10).

### 2.2. Preparation of Hydrogen-Rich Preservation Solution

**Figure 1:**
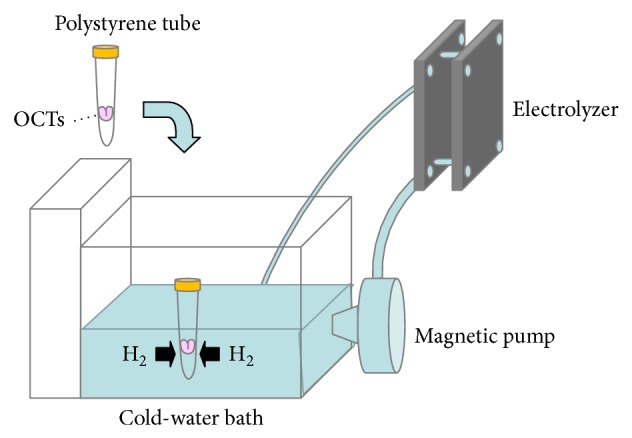
Hydrogen electrolyzer system used to introduce hydrogen into the preservation solution used for long-term cold storage of osteochondral tissue (OCT). The MiZ hydrogen electrolyzer system consists of an electrolyzer and a water bath connected to a refrigeration system. OCT in preservation solution within a hydrogen-permeable polystyrene tube is immersed in the cold-water bath for the introduction of hydrogen.

Hydrogen-rich preservation solution was prepared using a nondestructive hydrogen dissolver (MiZ Co., Ltd.) in a 4°C cold room as schematically outlined in Figure 1. Water circulating between the electrolyzer and water tank was electrolyzed periodically and stably saturated with hydrogen. OCTs samples were immersed in DMEM or UW solution in 15 mL polystyrene tubes, which were then placed in the cold-water bath of the electrolyzer system for 3 weeks. The hydrogen concentration in the preservation solution was estimated periodically using methylene blue-colloidal platinum catalyst reagents (MiZ Co., Ltd.), as previously described [18].

### 2.3. Measurement of Cell Viability in Preserved OCTs

We performed cell viability analysis after 21 days of preservation at 4°C because the rat OCT previously exhibited markedly reduced viability after this time point [19]. The cell viability of OCT samples was estimated by the water-soluble tetrazolium (WST) assay using a commercial WST kit (Cell Count Reagent SF; Nacalai Tesque, Kyoto, Japan), as previously described [19]. Nonpreserved (baseline group) and cold-preserved OCT samples with or without hydrogen supplementation were incubated at 37°C for 2 h in 3 mL culture medium containing 10% WST assay reagent. After incubation, the culture supernatant was transferred to 96-well plates, and the absorbance of each well was measured at 450 nm using a SpectraFluor Plus multiple plate reader (Tecan, Männedorf, Switzerland). The absorbance of ten OCT samples was averaged for each treatment group, and cell viability was then calculated using a standard curve of dye absorbance versus OCT sample quantity. The cell viability was expressed relative to the absorbance of one nonpreserved OCT sample (100%) and non-OCT-containing control sample (0%) [19, 20].

### 2.4. Histological Assessment

The assessment of morphologic features of cells in the articular cartilage of OCT samples was performed after the measurement of cell viability. OCT samples were fixed in a 4% paraformaldehyde phosphate buffer solution for 48 h at 4°C and were then decalcified in a 3 mol/L EDTA solution for 2 weeks at 4°C. After embedding the samples in paraffin, 3 *μ*m thick sagittal sections of the patellar groove of femoral condyles were prepared and then stained with hematoxylin and eosin. The thickest site of articular cartilage in the noncalcified zones was readily observed at 200x magnification using a light microscope. Each set of ten tissue specimens prepared from OCT samples preserved in preservation solution with or without hydrogen was assessed using a computer-assisted image filing system (Flovel, Tokyo, Japan). To determine the proportion of degenerative chondrocytes, the total number of chondrocytes in noncalcified zones was counted, and the number of chondrocytes with normal morphologic features (normal grade), mild pyknotic and irregular nuclei (mild grade), and severe pyknotic nuclei and eosinophilic shrunken cytoplasm (severe grade) [19] was determined and divided by the total number of chondrocytes.

### 2.5. Real-Time PCR

Total RNA was extracted from harvested OCT samples using TRIzol (Invitrogen, Carlsbad, CA) according to the manufacturer's instructions and was used as template for first-strand cDNA synthesis using SuperScript III RT (Invitrogen). The PCR reaction mixture consisted of 2 *μ*L cDNA, the specific primer set (0.2 *μ*M final concentration), and 12.5 *μ*L SYBR* Premix Ex Taq* (Takara, Kyoto, Japan) in a final volume of 25 *μ*L. The sequences of the PCR primer pairs are listed in Table 1. Quantitative PCR was performed using a Real-Time PCR Detection System (CFX-96; Bio-Rad, CA, USA). The PCR cycle parameters consisted of an initial denaturation at 95°C for 1 min, followed by 40 cycles of 95°C for 5 sec, and 60°C for 30 sec. mRNA expression was normalized to the levels of GAPDH mRNA.

## 3. Results

### 3.1. Hydrogen Concentration in Preservation Solution Submersed in a Hydrogen-Water Bath

**Figure 2:**
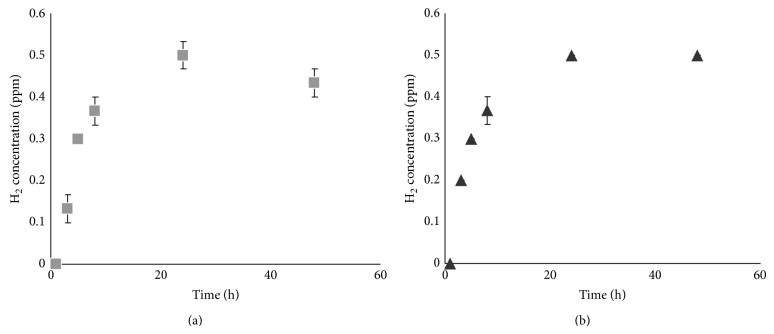
Measurement of hydrogen concentration in OCT preservation solution after hydrogen electrolyzer treatment. (a) DMEM. (b) UW solution. Each data point represents the mean and error bars show the SE.

The hydrogen concentrations of DMEM and UW preservation solutions containing OCT samples in polystyrene tubes submersed in the water bath of the hydrogen electrolyzer system were measured periodically over a 48 h period (Figure 2). The hydrogen concentration in both DMEM and UW solution increased rapidly, finally reaching a plateau of 0.5 ppm after 24 h of incubation (Figures 2(a) and 2(b)). No differences in the hydrogen concentrations of DMEM and UW solution were detected at any measurement time point. In addition, hydrogen was undetectable in DMEM and UW solution placed in the cold-water bath under nonelectrolyzing conditions.

### 3.2. Effect of Hydrogen on OCT Viability after Cold Preservation

**Figure 3:**
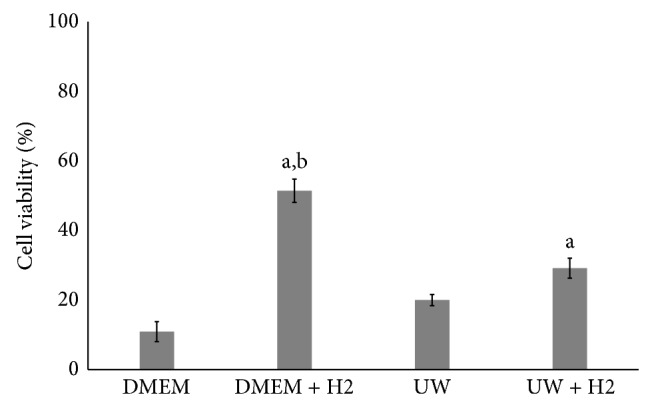
Effect of hydrogen on cell viability of OCT after cold preservation for 3 weeks in DMEM and UW solution. Data are presented as the mean ± SE (*n* = 10). ^a^Significant difference between the H2-treated and H2 nontreated groups (*P* < 0.05). ^b^Significantly different between the UW with H2 and DMEM H2 groups (*P* < 0.05).

As determined using a WST assay, OCT samples preserved in DMEM and UW solution supplemented with hydrogen had significantly higher cell viability after 21 days at 4°C than those stored in solution alone (*P* < 0.05; Figure 3). However, the cell viability of OCTs stored DMEM supplemented with hydrogen was significantly higher than that of OCTs in hydrogen-supplemented UW solution (*P* < 0.05). The mean percentages of cell viability of OCT samples maintained in DMEM and UW solution without hydrogen were 10.9% and 20.0%, respectively, whereas those for samples preserved in DMEM and UW solution supplemented with hydrogen were 51.4% and 29.1%, respectively.

### 3.3. Histomorphometric Analysis

**Figure 4:**
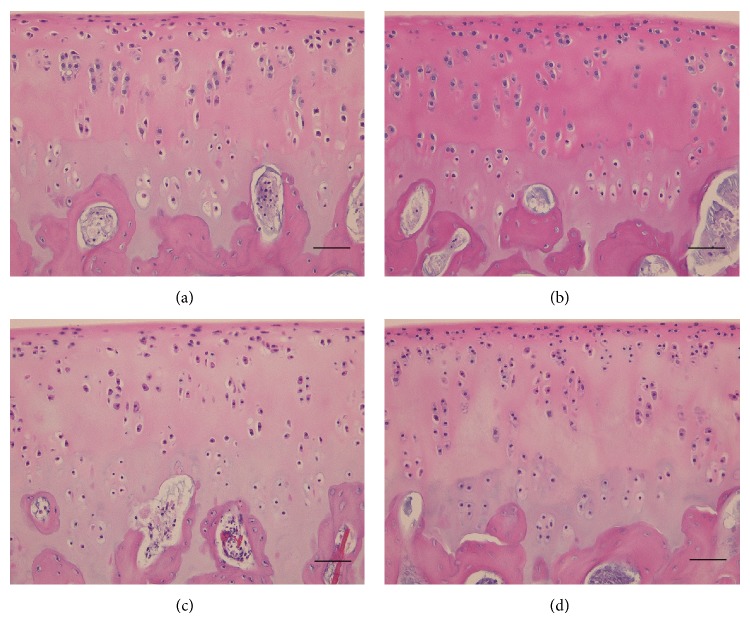
Representative hematoxylin and eosin-stained tissue sections of OCTs cold-preserved in the presence and absence of supplemental hydrogen. Histological analysis of OCTs was performed after 3 weeks of preservation in DMEM (a), DMEM with H2 (b), UW solution (c), and UW solution with H2 (d). Scale bar, 50 *μ*m.

To determine the proportion of degenerative chondrocytes in OCTs after long-term cold storage, sections of OCTs after 21 days of cold preservation in DMEM and UW solution supplemented with and without hydrogen were hematoxylin and eosin stained and analyzed by light microscopy (Figure 4). After 21 days at 4°C, OCT samples preserved in DMEM and UW solution supplemented with hydrogen had significantly more normal-grade and fewer severe-grade chondrocytes compared with those stored without hydrogen supplementation (*P* < 0.05; Table 2). The mean proportions of normal-grade chondrocytes in OCTs preserved in DMEM and UW solution alone were 9.0% and 0.8%, respectively, whereas those in DMEM and UW solution supplemented with hydrogen were 10.4% and 6.1%, respectively. The mean proportions of severe-grade chondrocytes in OCTs preserved in DMEM and UW solution alone were 47.0% and 94.2%, respectively, whereas those in DMEM and UW solution supplemented with hydrogen were only 11.8% and 47.0%, respectively.

### 3.4. Real-Time PCR Analysis

**Figure 5:**
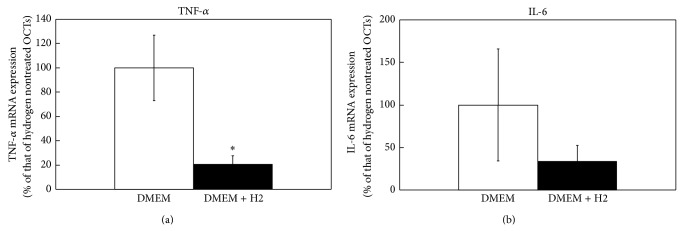
Real-time PCR analysis of OCTs cold-preserved in the presence and absence of supplemental hydrogen. Expression of TNF-*α* (a) and IL-6 mRNAs (b) in H2-treated and nontreated DMEM groups. ∗ indicates a statistically significant difference between the H2-treated and nontreated DMEM groups. All data are shown as the mean ± SE (*n* = 6).

To determine the mechanism of improvement of OCT viability after long-term cold storage with hydrogen, OCTs in DMEM supplemented with and without hydrogen were analyzed by real-time PCR, which showed that the expression of the TNF-*α* gene was significantly decreased in OCTs stored in DMEM supplemented with hydrogen compared to those in DMEM alone (Figure 5(a)). Similarly, expression of the IL-6 gene was decreased in OCTs stored in DMEM supplemented with hydrogen compared to those in DMEM alone, although the difference was not statistically significant (Figure 5(b)).

## 4. Discussion

In this study, hydrogen supplementation of storage medium significantly improved the cell viability of OCTs during long-term (21 day) cold preservation. Storage of OCTs in DMEM containing hydrogen at 4°C was associated with the most optimal maintenance of chondrocytes with respect to viability and morphology in comparison with UW solution and nonsupplemented conditions. Our findings show that OCTs cold-preserved in DMEM supplemented with hydrogen are a promising material for the efficient repair of large cartilage defects in the clinical setting.

Chondrocyte viability of allografts, particularly at the tissue surface, during cold storage appears to be a strong determinant of tissue properties and long-term repair efficacy after transplantation [2]. Therefore, storage conditions that preserve surface viability are essential for improving the quality of available graft material, such as OCT. In the present study, hydrogen gas was evaluated as a potential material for improving OCT quality during long-term storage due to its antiapoptotic and anti-inflammatory effects associated with its antioxidant properties [9, 12]. In addition, recent studies have shown that hydrogen-supplemented organ preservation solution, such as UW solution and Celsior solution, markedly reduces cell damage during organ preservation and subsequent transplantation [14, 17]. For example, Noda et al. [14] reported that the cold preservation of cardiac grafts in hydrogen-rich Celsior solution reduced myocardial injury due to cold ischemia and reperfusion, while Abe et al. [17] demonstrated that the cold preservation of kidneys in hydrogen-rich UW solution improves renal function and prolongs recipient survival rate compared with UW solution alone. Based on the above-described findings, we speculated that hydrogen-rich UW solution would be the most optimal for the long-term preservation of OCTs, because UW solution contains several antioxidants, including glutathione and allopurinol. However, hydrogen-supplemented culture medium, DMEM, had a much more profound effect on all of the parameters of tissue viability for OCTs examined in this study. Although UW solution is generally used for the short-term (1 to 3 days) cold preservation of organs, we previously showed that OCTs cold-preserved in UW solution supplemented with allogenic serum for 3 weeks had improved viability compared to those preserved in nonsupplemented UW solution [20]. The glutathione present in UW solution has a half-life of approximately one day [21]; therefore, serum-free UW solutions are not expected to provide superior preservation benefits compared to culture media. DMEM is approved for the storage and preservation of human transplant tissue, including OCT, in the clinical setting. Here, we demonstrated that the supplementation of DMEM with hydrogen improves the cell viability of OCTs without the necessity of added serum. Thus, DMEM supplemented with hydrogen is a promising solution for the cold preservation of OCTs in the clinical setting.

The inflammatory cytokine TNF-*α* contributes to chondrocyte apoptosis and cartilage degeneration [22, 23]. TNF-*α* is increased during the cold preservation of OCT and the addition of TNF-*α* inhibitor to preservation solution was shown to improve the viability of OCT [3]. Recent studies have also demonstrated that hydrogen supplementation attenuates TNF-*α* expression during organ preservation [14, 17]. Here, we found that supplementation of the preservation solution with hydrogen markedly reduces TNF-*α* expression of OCT. Although further studies are needed to determine the underlying mechanisms, our findings suggest that hydrogen improves OCT viability through the inhibition of TNF-*α* production.

To prevent marked decline in the cell viability of graft material and organs, several antioxidant and anti-inflammatory agents, such as epigallocatechin-3-O-gallate, tumor necrosis factor-alpha inhibitor, and glutathione, have been added to preservation solutions [3, 20, 24]. However, because drug efficacy gradually decreases as a function of the drug's half-life, preservation solutions require the continuous addition of cytoprotective agents during tissue storage. However, the cost and risk of contamination increase each time the preservation solution is changed. The tissue graft preservation method described here has the potential to provide long-term antioxidant effects and maintain a high level of cell viability and is therefore suitable for the storage of OCTs used in allografting procedures. Our strategy allows for the continuous delivery of hydrogen to preservation solution in a simple and cost-effective manner that maintains the viability and safety of OCTs during cold preservation.

## 5. Conclusions

The continuous delivery of hydrogen to culture medium improved OCT viability during cold preservation. The present findings suggest that OCTs preserved in hydrogen-rich culture media are a promising material for cartilage repair in the clinical setting.

## Figures and Tables

**Table 1:** Sequences of the primers used in this study.

Gene	Direction	Primer sequence (5′-3′)	Product size (bp)
TNF-*α*	F	CTCTTCTCATTCCCGCTCGT	104
	R	GGGAGCCCATTTGGGAACTT	

IL-6	F	CCAGTTGCCTTCTTGGGACT	224
	R	TCTGACAGTGCATCATCGCT	

GAPDH	F	TGCCACTCAGAAGACTGTGG	129
	R	TTCAGCTCTGGGATGACCTT	

**Table 2:** Proportion of each grade of chondrocyte in preserved OCT samples.

Storage condition	Normal (%)	Mild (%)	Severe (%)
DMEM	9.0 ± 3.3	40.1 ± 7.4	51.1 ± 10.1
DMEM + H2	10.4 ± 2.9	77.7 ± 4.2^a,b^	11.8 ± 5.0^a,b^
UW	0.8 ± 0.5	5.1 ± 1.6	94.2 ± 2.0
UW + H2	6.1 ± 0.7^a^	46.7 ± 2.5^a,b^	47.0 ± 2.6^a,b^

Data are presented as the mean ± SE (*n* = 10). ^a^Significant difference between the H2-treated and H2 nontreated groups (*P* < 0.05). ^b^Significant difference between the UW H2 and DMEM H2 groups (*P* < 0.05).
